# Phytocannabinoids Act Synergistically with Non-Steroidal Anti-Inflammatory Drugs Reducing Inflammation in 2D and 3D In Vitro Models

**DOI:** 10.3390/ph15121559

**Published:** 2022-12-14

**Authors:** Ajjampura C. Vinayaka, Nurit Shalev, Seegehalli M. Anil, Sudeep Tiwari, Navin Kumar, Eduard Belausov, Karthik Ananth Mani, Guy Mechrez, Hinanit Koltai

**Affiliations:** 1 Institute of Plant Science, Agriculture Research Organization, Volcani Institute, Rishon LeZion 7505101, Israel; 2 Institute of Postharvest and Food Sciences, Agriculture Research Organization, Volcani Institute, Rishon LeZion 7505101, Israel; 3 Institute of Biochemistry, Food Science and Nutrition, The Robert H. Smith Faculty of Agriculture, Food and Environment, The Hebrew University of Jerusalem, Rehovot 7610001, Israel

**Keywords:** cannabinoids, lung, inflammation, interleukin, chemokine, epithelial cells, macrophages, corticosteroids, NSAID

## Abstract

Lung inflammation is associated with elevated pro-inflammatory cytokines and chemokines. Treatment with FCBD:std (standard mix of cannabidiol [CBD], cannabigerol [CBG] and tetrahydrocannabivarin [THCV]) leads to a marked reduction in the inflammation of alveolar epithelial cells, but not in macrophages. In the present study, the combined anti-inflammatory effect of FCBD:std with two corticosteroids (dexamethasone and budesonide) and two non-steroidal anti-inflammatory drugs (NSAID; ibuprofen and diclofenac), was examined. Enzyme-linked immunosorbent assay (ELISA) was used to determine protein levels. Gene expression was determined by quantitative real-time PCR. Inhibition of cyclo-oxygenase (COX) activity was determined in vitro. FCBD:std and diclofenac act synergistically, reducing IL-8 levels in macrophages and lung epithelial cells. FCBD:std plus diclofenac also reduced *IL-6*, *IL-8* and *CCL2* expression levels in co-cultures of macrophages and lung epithelial cells, in 2D and 3D models. Treatment by FCBD:std and/or NSAID reduced *COX-1* and *COX-2* gene expression but not their enzymatic activity. FCBD:std and diclofenac exhibit synergistic anti-inflammatory effects on macrophages and lung epithelial cells, yet this combined activity needs to be examined in pre-clinical studies and clinical trials.

## 1. Introduction

An intense host inflammatory response of the lung to infection often leads to the development of intra-alveolar, interstitial fibrosis and alveolar damage [1]. Acute respiratory distress syndrome (ARDS) is the leading cause of mortality in Coronavirus Disease 2019 (COVID-19) caused by coronavirus SARS-CoV-2 [2]. Lung acute immune response involves a cytokine storm leading to a widespread lung inflammation with elevated pro-inflammatory cytokines and chemokines, mainly tumor necrosis factor alpha (TNFα), interleukin (IL)-6, IL-8 and C-C Motif Chemokine Ligand 2 (CCL2) [3,4,5]. During lung inflammation, monocyte-derived macrophages are activated and play a major pro-inflammatory role [6] by releasing pro-inflammatory cytokines such as IL-6 and IL-8 [7]. Additionally, in coronavirus-induced severe acute respiratory syndrome (SARS), lung epithelial cells also release pro-inflammatory cytokines including IL-8 and IL-6 [8]. 

Lung inflammation is usually treated by corticosteroid-based medications, such as budesonide [9]. Dexamethasone too has anti-inflammatory activity in lung epithelial cells [10]. Additionally, Carbonic Anhydrase Inhibitor (CAI)—Nonsteroidal-Anti-Inflammatory Drug (NSAID) hybrid compounds have been demonstrated in vivo to be new anti-inflammatory drugs for treating chronic lung inflammation [11].

*Cannabis sativa* is broadly used for the treatment of several medical conditions. Strains of cannabis produce more than 500 different constituents, including phytocannabinoids, terpenes and flavonoids [12,13,14]. Phytocannabinoids were shown to influence macrophage activity and to alter the balance between pro- and anti-inflammatory cytokines, and thus have some immunomodulation activity [15,16].

For example, Δ9-tetrahydrocannabinol (THC) inhibits macrophage phagocytosis by 90% [17], and in lipopolysaccharide-activated macrophages, Δ9-tetrahydrocannabivarin (THCV) inhibited IL-1β protein levels [18]. Cannabidiol (CBD) was shown to reduce the production of IL-6 and IL-8 in rheumatoid arthritis synovial fibroblasts [19] and was suggested to be added to anti-viral therapies to alleviate COVID-19-related inflammation [20]. 

Previously, we showed that FCBD:std treatment, which is based on a mixture of phytocannabinoids (CBD, cannabigerol [CBG] and THCV; composition is originated from a fraction of *C. sativa* var. ARBEL [indica] extract), leads to a marked reduction in the level of inflammation in alveolar epithelial cells but not in macrophages [21]. Hence, to explore a plausible approach for reducing inflammation also in macrophages, we sought to examine the combinatory anti-inflammatory effect of FCBD:std with two steroid-based and two NSAID anti-inflammatory pharmaceutical drugs. 

## 2. Results

### 2.1. Determining the Effect of FCBD:std and Anti-Inflammatory Drug Combinations on Macrophage Inflammation

We have examined the ability of FCBD:std to affect the anti-inflammatory activity of ibuprofen, budesonide, dexamethasone and diclofenac in differentiated monocytes (macrophages, KG1 cells), determined by IL-8 levels. 

**Figure 1:**
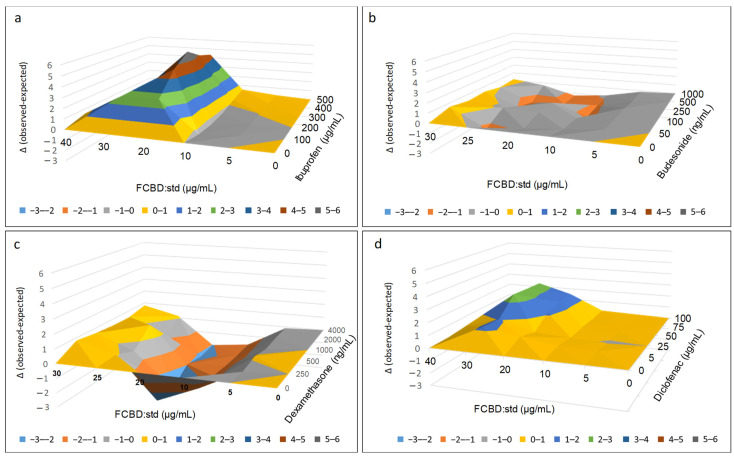
Synergistic interactions between FCBD:std with (**a**) ibuprofen, (**b**) budesonide, (**c**) dexamethasone or (**d**) diclofenac on KG1 cell IL-8 levels following combined treatments. The Bliss independence-drug interaction model was used to calculate synergy. Synergy is determined when the experimental value of IL-8 reduction is higher than the calculated value. In the Y axis is the delta between the experimental and the calculated values. In Supplementary Table S2, different letters indicate significantly different levels of the experimental (observed) values from all pairs’ combinations according to Tukey–Kramer honest significant difference test (*n* = 3; HSD; *p* ≤ 0.05).

In contrast to FCBD:std, which led to an increased level of IL-8 secretion, treatment with 20 µg/mL of FCBD:std and 300, 400 or 500 µg/mL of ibuprofen, resulted in an anti-inflammatory activity that was 3.6, 3.6 and 5.7-fold higher, respectively, than ibuprofen alone at these concentrations (Supplementary Table S1a). Based on the Bliss independence drug interaction model, significant synergy (identified once the observed [experimental] value of IL reduction is higher than the expected [calculated] value) was apparent with 20 or 30 µg/mL of FCBD:std and 100–500 µg/mL of ibuprofen (Figure 1a; Supplementary Table S1a).

Treatment with 30 µg/mL FCBD:std and 50 ng/mL budesonide showed a 2.0-fold higher activity than budesonide alone at this concentration (Supplementary Table S1b). However, the synergy (i.e., the delta value of the experimental [observed] value of IL-8 reduction minus the calculated [expected] value of the combination) between FCBD:std and budesonide was not significantly different in comparison to treatment with either FCBD:std or budesonide alone (Figure 1b; Supplementary Table S1b). Other combinations of FCBD:std and budesonide led to significant inhibition of activity (e.g., 25 µg/mL of FCBD:std and 500 ng/mL budesonide; Figure 1b; Supplementary Table S1b). 

Treatment with 30 µg/mL FCBD:std and 2000 ng/mL dexamethasone resulted in activity that was 3.7-fold higher than dexamethasone alone (Supplementary Table S1c). However, when compared to treatment with each component independently, the combined treatment activity was not significantly higher (Figure 1c; Supplementary Table S1c). Moreover, inhibition rather than activity enhancement was apparent in other combinations of FCBD:std and dexamethasone (e.g., 20 µg/mL and 250 ng/mL of FCBD:std and dexamethasone, respectively; Figure 1c; Supplementary Table S1c). 

Treatment with 30 µg/mL FCBD:std and 50, 75 or 100 µg/mL of diclofenac showed 1.2, 2.0 and 1.9-fold higher activity than diclofenac alone, respectively (Supplementary Table S1d). The synergy between FCBD:std and diclofenac was apparent mainly at 30 µg/mL FCBD:std and 50–100 µg/mL diclofenac (Figure 1d; Supplementary Table S1d).

### 2.2. Determining the Effect of FCBD:std and Anti-Inflammatory Drug Combinations on Lung Epithelial Cell Inflammation

We examined the ability of FCBD:std to increase the anti-inflammatory activity of ibuprofen, budesonide, dexamethasone and diclofenac in A549 cells, determined by IL-8 levels. 

**Figure 2:**
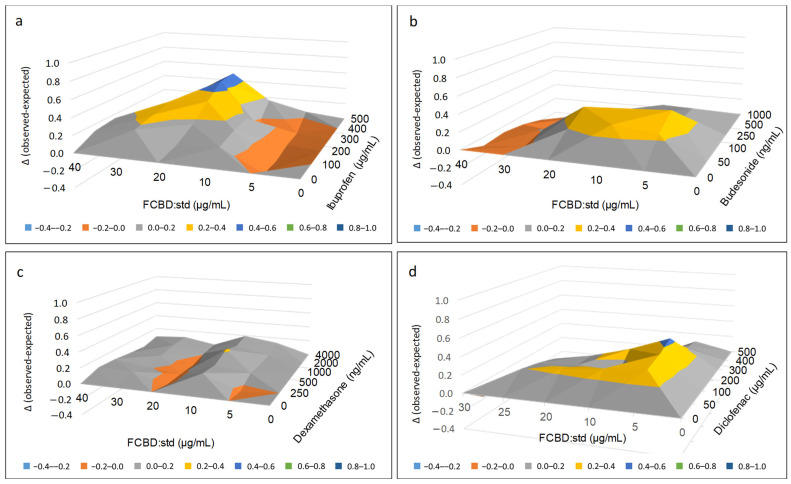
Synergistic interactions between FCBD:std with (**a**) ibuprofen, (**b**) budesonide, (**c**) dexamethasone or (**d**) diclofenac on IL-8 level of A549 cells following combined treatments. The Bliss independence-drug interaction model was used to calculate synergy. Synergy is determined when the experimental value of IL-8 reduction is higher than the calculated value. In the Y axis is the delta between the experimental and the calculated values. In Supplementary Table S2, different letters indicate significantly different levels of the experimental (observed) values from all pairs’ combinations according to Tukey–Kramer honest significant difference test (*n* = 3; HSD; *p* ≤ 0.05).

The combined treatment with FCBD:std (20 µg/mL) and ibuprofen at 400 and 500 µg/mL was 2.0 or 2.2-fold more active than FCBD:std or ibuprofen alone, respectively (Supplementary Table S3a). FCBD:std and ibuprofen treatments were significantly synergistic, especially at these concentrations (Figure 2a; Supplementary Table S3a). 

Treatments with 5 or 20 µg/mL of FCBD:std and 100 ng/mL budesonide resulted in activity that was 1.8 and 2.0-fold higher, respectively, than budesonide alone. The concentration of 10 µg/mL of FCBD:std with 50 or 100 ng/mL budesonide resulted in 1.5 and 1.6-fold higher activity, respectively, than budesonide alone (Supplementary Table S3b). Similarly, treatment with FCBD:std and budesonide at these concentrations led to significant synergy (Figure 2b; Supplementary Table S3b).

Treatments with FCBD:std and dexamethasone lead to only a minor increase in activity (Supplementary Table S3c), significant synergy was detected mainly at 10 µg/mL FCBD:std combined with 1000 ng/mL dexamethasone (Figure 2c; Supplementary Table S3c). 

Treatments with 5 µg/mL FCBD:std and 50, 100, 200, and 300 µg/mL of diclofenac showed 1.4, 1.5, 1.8 and 1.5-fold higher activity than diclofenac alone, respectively (Supplementary Table S3d). Synergy was significant at these concentrations and 10 µg/mL FCBD:std with 50 or 200 µg/mL diclofenac (Figure 2d; Supplementary Table S3d).

### 2.3. Determining the Effect of Treatments in Co-Cultures of A549 and Differentiated KG1 Cell Lines

#### 2.3.1. Determining the Effect of Treatments on IL-8 Protein Levels in Co-Cultures of A549 and Differentiated KG1 Cell Lines

**Figure 3:**
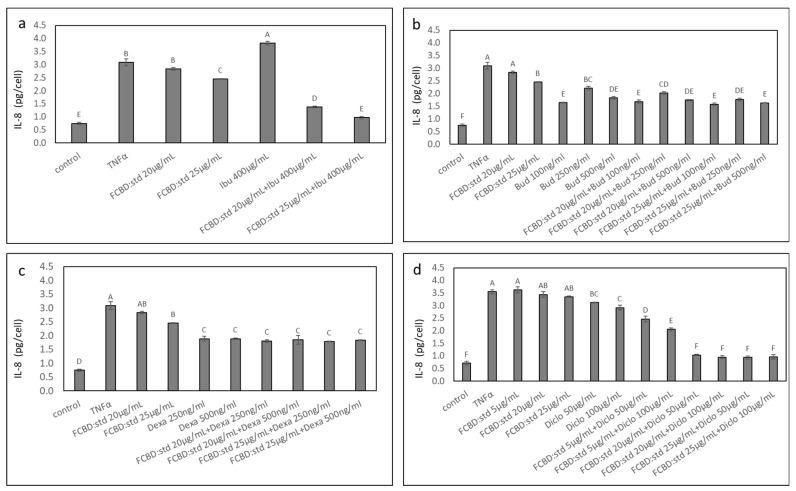
Effect of treatments with FCBD:std and (**a**) ibuprofen (Ibu), (**b**) budesonide (Bud), (**c**) dexamethasone (Dexa) or (**d**) diclofenac (Diclo) on IL-8 levels in co-cultures of A549 and differentiated KG1 cell lines. Control is vehicle control (1% methanol, 0.5% DMSO, 0.2% methanol + 0.5% DMSO and 1% DMSO for Ibu, Bud, Dexa and Diclo, respectively). TNFα is solvent control + TNFα. Means ± SE (*n* = 3) are shown. Levels of means with different letters are significantly different from all pairs’ combinations according to Tukey–Kramer honest significant difference (HSD; *p* ≤ 0.05).

Subsequently, we determined the anti-inflammatory activity of synergistic treatments based on their effect in monoculture, in co-culture of A549 and macrophages (differentiated KG1). FCBD:std had only minor anti-inflammatory activity; ibuprofen at 400 µg/mL even increased IL-8 secretion per cell (Figure 3a). Treatments with 20 or 25 µg/mL FCBD:std and 400 µg/mL ibuprofen reduced IL-8 substantially (Figure 3a). This activity was 2.4 and 2.2-fold greater than the individual compounds (for 25 µg/mL FCBD:std and 400 µg/mL ibuprofen, respectively; Figure 3a). 

Dexamethasone or budesonide had significant anti-inflammatory activity. Nevertheless, combinations of FCBD:std with dexamethasone or budesonide did not show any additive anti-inflammatory effect (Figure 3b,c).

All treatments with combinations of 20 or 25 µg/mL FCBD:std and 50 or 100 µg/mL diclofenac reduced IL-8 substantially (Figure 3d). For the 20 µg/mL FCBD:std and 50 or 100 µg/mL diclofenac co-treatment, this activity was 2.9-fold greater than that of the individual compounds only (Figure 3d). For the 25 µg/mL FCBD:std and 50 or 100 µg/mL diclofenac co-treatment, this activity was 3.2 and 2.9-fold greater than that of the individual compounds, FCBD:std or diclofenac, respectively (Figure 3d). 

#### 2.3.2. Determining the Effect of Treatments on IL-6 Protein Levels in Co-Cultures of A549 and Differentiated KG1 Cell Lines

**Figure 4:**
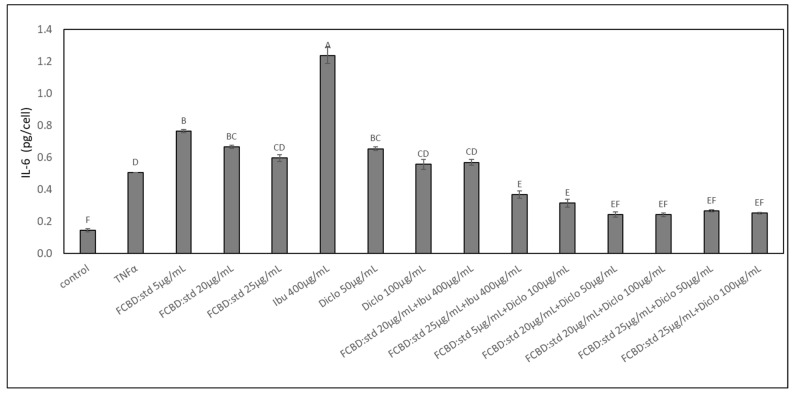
The effect of treatments with FCBD:std and diclofenac (Diclo) or ibuprofen (Ibu) on IL-6 levels in co-cultures of A549 and differentiated KG1 cell lines. Control is vehicle control (1% methanol; 1% DMSO vehicle control is not significantly different from 1% methanol, not shown). TNFα is solvent control + TNFα. Means ± SE (*n* = 3) are shown. Levels of means with different letters are significantly different from all pairs’ combinations according to Tukey–Kramer honest significant difference (HSD; *p* ≤ 0.05).

The activity of synergistic treatments, previously determined based on their effect in monocultures, with combinations of FCBD:std and ibuprofen or diclofenac were examined on IL-6 protein levels in co-cultures of A549 and macrophages. Treatments with 5 or 20 µg/mL FCBD:std led to a minor increase in IL-6 levels (in agreement with [21]); ibuprofen at 400 µg/mL led to a substantial increase in IL-6 secretion per cell, and treatment with diclofenac did not reduce IL-6 secretion levels (Figure 4). Treatment with 20 µg/mL FCBD:std and 400 µg/mL ibuprofen did not reduce IL-6 levels, and treatment with 25 µg/mL FCBD:std and 400 µg/mL ibuprofen led to a slight reduction in IL-6 levels (Figure 4). However, treatments with all examined combinations of FCBD:std (5, 20 or 25 µg/mL) and diclofenac (50 or 100 µg/mL) led to a substantial reduction of IL-6 levels in the co-culture (Figure 4). 

#### 2.3.3. Determining the Effect of Treatments on IL-6, IL-8 and CCL-2 Gene Expression Levels in Co-Cultures of A549 and Differentiated KG1 Cell Lines

**Figure 5:**
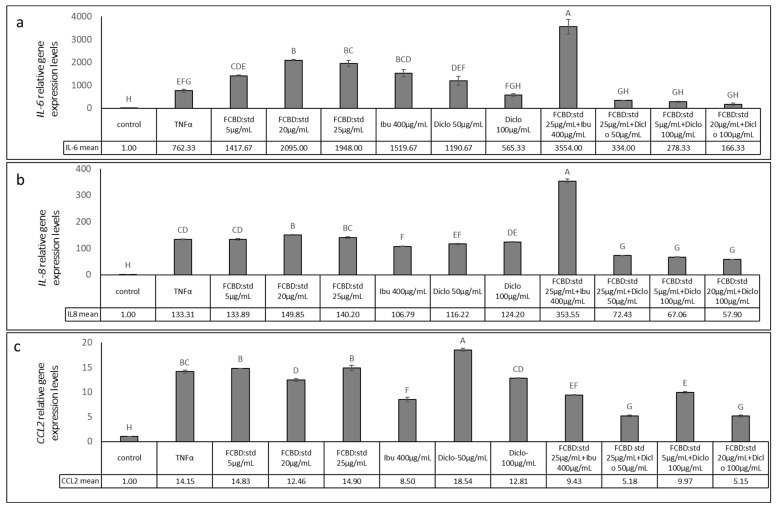
Determination of the RNA steady-state level using quantitative PCR-based in co-culture of A549 and differentiated KG1 cell line of (**a**) *IL-6*, (**b**) *IL-8* or (**c**) *CCL2* after treatment for 2 h with FCBD:std or ibuprofen (Ibu) or diclofenac (Diclo) and their combinations, relative to control. Quantitative PCR determined gene transcript values as a ratio between the target gene versus β-actin as a reference gene. Using the 2^ΔΔCt^ method, values were calculated relative to the average expression of target genes in treated versus control. Control (1% methanol) treatment served as solvent (vehicle) control. TNFα is solvent control+TNFα. Means ± SE (*n* = 3) are shown. Levels of means with different letters are significantly different from all pairs’ combinations according to Tukey–Kramer honest significant difference (HSD; *p* ≤ 0.05).

We subsequently examined *IL-6* and *IL-8* gene expression levels following the most effective synergistic treatments determined so far—combinations of FCBD:std and ibuprofen or diclofenac—in the co-culture of A549 and macrophages. Expression of both *IL-6* and *IL-8* increased with TNFα treatment (Figure 5). 

In accordance with the IL-6 protein levels, FCBD:std treatment of 5 and 20 µg/mL led to a minor increase in *IL-6* gene expression (in agreement with [21]); ibuprofen at 400 µg/mL also led to a substantial increase in *IL-6* expression per cell whereas diclofenac at 100 µg/mL led to a reduction in IL-6 expression (Figure 5a). In contrast to the IL-6 protein results, the combination of 25 µg/mL FCBD:std and 400 µg/mL ibuprofen led to a large increase in *IL-6* expression levels (Figure 5a). However, and in agreement with the IL-6 protein data, all examined combinations of FCBD:std (5, 20 or 25 µg/mL) and diclofenac (50 or 100 µg/mL) led to a substantial reduction of *IL-6* expression levels in the co-culture. Downregulation of *IL-6* gene expression was higher in these co-treatments compared to diclofenac only (Figure 5a). 

When the *IL-8* expression was examined, treatments with FCBD:std, ibuprofen or diclofenac had only minor effects on *IL-8* expression levels (Figure 5b). The combined treatments with FCBD:std and diclofenac were the most effective, substantially reducing *IL-8* gene expression (Figure 5b).

Treatments with FCBD:std or diclofenac had only minor effects on *CCL2* expression. Diclofenac at a 100 µg/mL concentration even increased *CCL2* expression to some extent (Figure 5c). Ibuprofen reduced *CCL2* expression level considerably; however, the combined treatments of FCBD:std and diclofenac were the most effective, substantially reducing *CCL2* gene expression level (Figure 5c).

#### 2.3.4. Determining the Effect of Treatments on COX-1 and COX-2 Gene Expression in Co-Cultures of A549 and Differentiated KG1 Cell Lines

**Figure 6:**
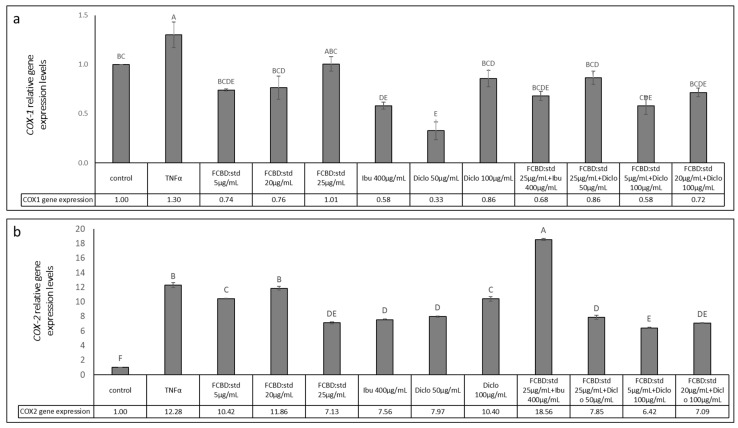
Determination of the RNA steady-state level using quantitative PCR-based in co-culture of A549 and differentiated KG1 cell line of (**a**) *COX-1*, (**b**) *COX-2* after treatment with FCBD:std or ibuprofen (Ibu) or diclofenac (Diclo) and their combinations, for 2 h relative to control. Quantitative PCR determined gene transcript values as a ratio between the target gene versus β-actin as a reference gene. Using the 2^ΔΔCt^ method, values were calculated relative to the average expression of target genes in treated versus control. Control (1% methanol) treatment served as solvent (vehicle) control. TNFα is solvent control+ TNFα. Means ± SE (*n* = 3) are shown. Levels with different letters are significantly different from all pairs’ combinations by the Tukey–Kramer honest significant difference test (HSD; *p* ≤ 0.05).

We examined the level of *COX-1* and *COX-2* gene expression in the co-culture of A549 and macrophages following the most effective treatments by combinations of FCBD:std and ibuprofen or diclofenac. *COX-1* expression was substantially increased with TNFα treatment and reduced with the ibuprofen and diclofenac treatments. However, *COX-1* expression was not significantly further reduced in the combined treatments of FCBD:std and ibuprofen or diclofenac treatments (Figure 6a). 

*COX-2* expression was substantially increased with TNFα treatment and reduced with ibuprofen and diclofenac treatments (Figure 6b). Treatment with FCDB:std only at 5 µg/mL reduced *COX-2* expression to a minor extent. However, treatment with FCBD:std and ibuprofen led to a marked increase in *COX-2* expression. Treatment with FCBD:std and diclofenac substantially reduced *COX-2* expression, co-treatment by FCBD:std 5 µg/mL and diclofenac 100 µg/mL to a level lower than that by diclofenac only (Figure 6b). 

Ibuprofen and diclofenac substantially inhibited COX-1 and COX-2 enzyme activity. Nevertheless, FCBD:std did not inhibit their activity at the examined concentration (Supplementary Figure S1). 

### 2.4. Determining the Effect of Treatments in 3D Models of Co-Cultures of A549 and Differentiated KG1 Cell Lines 

#### 2.4.1. Establishing 3D Co-Cultures of A549 and Macrophages

**Figure 7:**
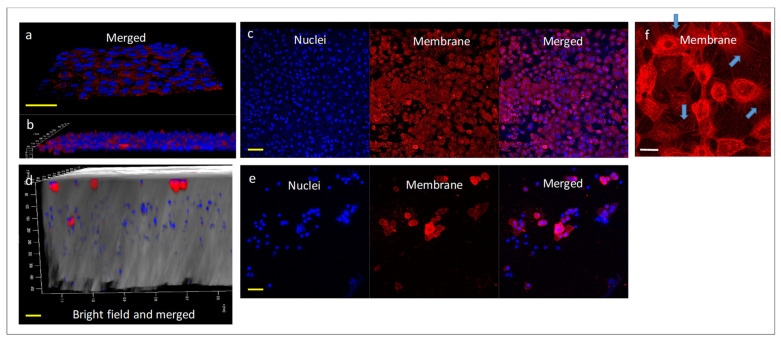
Confocal Images of 3D co-cultures. (**a**,**b**) 8-µm PET membrane seeded with A549 cells, angle (**a**) and side (**b**) views (Bar = 50 µm); (**c**) Monolayer of A549, top view (Bar = 50 µm); (**d**) ECM seeded with macrophages, side view (Bar = 50 µm); (**e**) Macrophages in ECM, top view (Bar = 50 µm). (**f**) Ciliated A549 cells on the membrane, arrows point to ciliate structures (Bar = 20 µm). Nuclei are stained by Hoechst (blue stain) and membrane by Dil perchlorate (red stain). Merged includes both nuclei and membrane staining—illustration of the 3D printed model in Supplementary Figure S2.

For the preparation of 3D co-cultures of A549 and macrophages that may bear a resemblance to alveoli, differentiated KG1 cells (i.e., macrophages) immersed in an extra-cellular matrix (ECM) were printed on an 8-µm polyethylene terephthalate (PET) membrane (Supplementary Figure S2). Next, the A549 culture was established on the 8-µm PET membrane to form a cell monolayer (Figure 7a–c). Macrophages were visualized in the ECM (Figure 7d,e) and medium (not shown), suggesting that macrophages were roaming the 3D structure. The A549 cells were ciliated (Figure 7f).

#### 2.4.2. Determining the Anti-Inflammatory Effect of Treatments on the 3D Co-Cultures

**Figure 8:**
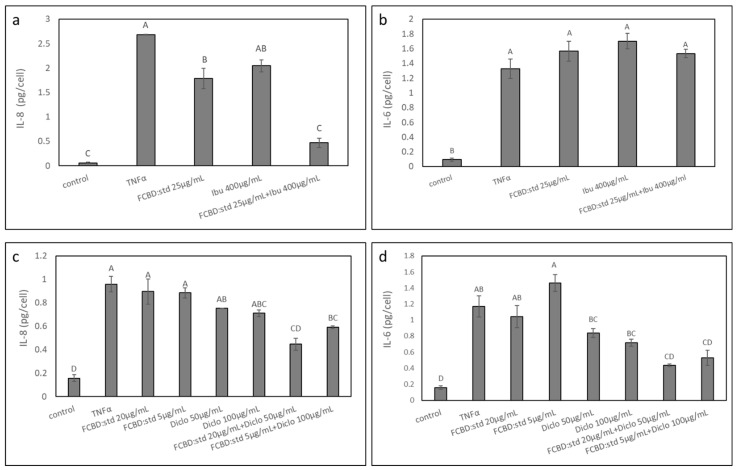
The effect of treatments with FCBD:std and/or ibuprofen (**a**,**b**) and FCBD:std and/or diclofenac (**c**,**d**) on IL-8 (**a**,**c**) or IL-6 (**b**,**d**) levels in a 3D co-culture model of A549 monolayer and differentiated KG1 cell lines in ECM. Control is vehicle control (1% methanol and 1% DMSO for Ibu and Diclo, respectively). TNFα is solvent control + TNFα. Means ± SE (*n* = 3) are shown. Levels of means with different letters are significantly different from all pairs’ combinations according to the Tukey–Kramer honest significant difference test (HSD; *p* ≤ 0.05).

The 3D structures of monolayer A459 and ECM macrophages were treated with FCBD:std and/or ibuprofen and FCBD:std and/or diclofenac. Only minor anti-inflammatory activity was recorded for the treatment with 25 µg/mL FCBD:std on IL-8 protein levels and no effect was recorded on IL-6 protein levels (Figure 8a,b). Treatment with ibuprofen only was inactive on both IL-8 and IL-6 levels (Figure 8a,b). However, treatment with a combination of 25 µg/mL FCBD:std and 400 µg/mL ibuprofen reduced IL-8 significantly (Figure 8a). Yet, this treatment did not reduce IL-6 levels in the 3D structures (Figure 8b). 

Treatment of the 3D co-cultures with 5 and 20 µg/mL FCBD:std did not substantially affect IL-8 or IL-6 levels (Figure 8c,d). Similarly, 50 or 100 µg/mL of diclofenac had only a minor effect on IL-8 or IL-6 levels (Figure 8c,d). However, once FCBD:std and diclofenac were combined to treat the 3D structures, there was a substantial reduction in both IL-8 and IL-6 levels (Figure 8c,d). 

## 3. Discussion

In the present work we show that FCBD:std and NSAIDs have synergistic anti-inflammatory effects on macrophage and lung epithelial cell models. Previously, we established the anti-inflammatory activity for FCBD:std, a mixture of CBD, CBG and THCV at determined ratios, in a lung epithelial (A549) cell line [21]. Nevertheless, FCBD:std treatment did not lead to a marked reduction in the gene expression and protein levels of IL-8 nor IL-6 in differentiated macrophages (KG1) cell lines at the examined concentrations [21]. 

In the present study, when monocultures of lung epithelial cells of macrophages were examined, FCBD:std increased the activity of NSAID (i.e., ibuprofen or diclofenac) by several fold (e.g., up to ~5.7 fold). FCBD:std also increased the activity of corticosteroids (i.e., budesonide or dexamethasone) to some extent. However, once the activity of the synergistic combinations between FCBD:std and drugs were examined in 2D lung epithelial cells and macrophage co-cultures, combinations of FCBD:std and diclofenac were highly effective in reducing IL-8 and IL-6 protein levels. These combinations also reduced *IL-6*, *IL-8* and *CCL2* gene expression levels. 

IL-6 and IL-8 are prominent cytokines in the ‘cytokine storm’ characteristic in severe COVID-19 patients and are secreted from alveolar epithelial cells during the disease [3]. CCL2 levels are also increased in bronchoalveolar fluids of severe COVID-19 patients; in these patients there is also an increased recruitment of monocytes into the lungs [6]. Our results suggest that the combined treatment of FCBD:std and diclofenac may reduce secretion of inflammatory cytokines associated with the disease, and potentially reduce the severity of the cytokine storm.

Synergistic anti-inflammatory activity was also recorded between FCBD:std and ibuprofen in IL-8 protein levels and *CCL2* gene expression levels. However, the increase in *IL-8* or *IL-6* gene expression in this treatment suggests that the anti-inflammatory activity inferred by the combined FCBD:std and ibuprofen treatment is only partial, and IL-8 or IL-6 protein levels may increase rather than reduce during a later stage of treatment. 

We also generated a 3D model consisting of a monolayer of ciliated lung epithelial cells, backed by ECM rich in collagen that contains macrophages with some infiltrating into the medium, to approximate the alveoli-cellular environment [22,23]. In this model, FCBD:std and ibuprofen combined treatment was ineffective in the 3D model. Nevertheless, there was a marked reduction of both IL-8 and IL-6 levels with the FCBD:std and diclofenac combined treatments; diclofenac or FCBD:std administered separately were almost inactive in IL reduction in this model. 

Diclofenac is a phenylacetic acid class NSAID. It has anti-inflammatory, analgesic, and antipyretic activity. It is widely used and available in oral or topical formulations to manage chronic inflammatory and degenerative joint diseases, including rheumatoid arthritis, osteoarthritis, extra-articular rheumatism and ankylosing spondylitis. It is also an alternative treatment for spinal and chronic central pain [24]. Ibuprofen is another NSAID, and for decades has been one of the world’s most widely used drugs to manage and treat mild to moderate pain, fever, inflammatory diseases, rheumatoid disorders, dysmenorrhea and osteoarthritis [25]. Diclofenac’s primary mode of action is inhibition of the activity of the COX-2 enzyme, and that of COX-1 to a lesser extent. This inhibition reduces the synthesis of pro-inflammatory and nociceptive prostaglandin E2 (PGE2) and thromboxane [24]. Ibuprofen inhibits both COX-1 and COX-2 activity [26,27].

We have found that the combined treatment of cannabis with NSAID led to reduced levels of *COX1* transcription in the lung epithelial cell and macrophage co-culture. However, diclofenac-only treatment was the most effective. Nevertheless, *COX-2* transcription was inhibited to the largest extent by the combined treatment of FCBD:std and diclofenac over all other examined treatments. Reduced *COX-2* expression indicates reduced prostaglandin synthesis and anti-inflammatory properties [28,29]. Additionally, in lung epithelial cells, reduction of *COX-2* expression is associated with reduced inflammation (e.g., ref. [30]) and up-regulation of *COX-2* and prostaglandin E2 (PGE2) are involved in lung inflammation [31,32]. However, COX-1 and COX-2 enzyme activity were not inhibited by FCBD:std. This contrasts with other studies suggesting cannabinoids reduce COX-2 activity [33,34]. Likely, there is no additive effect by FCBD:std on the COX inhibition activity of NSAIDs. 

Notably, both diclofenac and ibuprofen are associated with adverse effects. In the gastrointestinal system, ibuprofen may lead (dose-dependently and with prolonged use) to cellular disruptions and to compromise of mucosal integrity, causing higher permeability to, e.g., acid, which in turn may lead to ulcers [26]. Other adverse effects of ibuprofen depend on the dosage, concomitant medications, and patient population. Yet, ibuprofen has a comparatively low risk of cardiovascular adverse effects [26]. In contrast, diclofenac is associated with considerable, dose-dependent gastrointestinal, renal and cardiovascular adverse effects [35,36].

Moreover, diclofenac is an environmental hazard: its persistence in the environment and frequent occurrence in surface water (river, lake canal, estuary, and sea), groundwater and wastewater poses a serious threat to vultures, aquatic animals, plants and mammals. It is included in the Watch List of the EU Water Framework Directive [37,38]. Reduction in the use levels of NSAIDs may minimize treatment-adverse effects and environmental hazards. 

## 4. Material and Methods

### 4.1. Standard and Material Preparation and Use

The cannabinoid standard mix (FCBD:std) used in this study at a concentration of 1 mg/mL in methanol. FCBD:std included cannabidiol (CBD, 34011; Restek, PA, USA), cannabigerol (CBG, 34091; Restek) and tetrahydrocannabivarin (THCV, 34100; Restek). FCBD:std at the stock concentration of 60 µg/mL is the combination of CBD (62.2% *w*/*v*), CBG (4.04% *w*/*v*) and THCV (0.28% *w*/*v*) and was prepared by evaporating the solvent of CBD using N_2_ gas and mixing the dried CBD with CBG and THCV in complete DMEM growth media. Phorbol 12-myristate 13-acetate (PMA, P1585; Sigma Aldrich, St. Louis, MI, USA) was dissolved in DMSO as 5 µg/mL stock concentration. Ibuprofen (I4883; Sigma Aldrich, St. Louis, MI, USA) was dissolved in methanol as 50 mg/mL stock concentration. Diclofenac sodium (BP619; Sigma Aldrich, St. Louis, MI, USA) was dissolved in DMSO as 50 mg/mL stock concentration. Budesonide (B1157300-EP; Sigma Aldrich, St. Louis, MI, USA) was dissolved in DMSO as 200, 100, and 20 µg/mL stock concentrations. Dexamethasone (D4902; Sigma Aldrich, St. Louis, MI, USA) was dissolved in DMSO as 1000, 200, 100, and 20 µg/mL stock concentrations. TNFα (300-01A; PeproTech, Rocky Hill, NJ, USA) was dissolved in water as 100 µg/mL stock concentration. Analytical grade methanol was used at a final concentration of 0.8 to 0.2% (*v*/*v*) according to the indicated concentration used for each of the treatments. For vehicle control the highest methanol concentration in each experiment was used. Bio-grade Dimethyl Sulfoxide was used at a final concentration of 1 to 0.5% (*v*/*v*) according to the indicated concentration used for each of the treatments. Ultra-pure deionized water (MS grade) was used as received with no additional purification.

### 4.2. Cell Cultures

Macrophage cell line KG1 (ATCC^®^ CCL-246™, Manassas, VA, USA) was cultured in DMEM growth media supplemented with 20% FBS, 1% glutamic acid, 1% pen-strep and plasmocin. The lung cancer cell line A549 (ATCC^®^ CCL-185™) was cultured in DMEM (01-055-1A, Biological Industries, Beit Haemek, Israel) growth media supplemented with 10% FBS, 1% glutamic acid, 1% pen-strep and plasmocin. For the stimulating environment to induce KG1 cells differentiation, 10 ng/mL PMA in DMEM media supplemented with 10% FBS, 1% glutamic acid, 1% pen-strep and plasmocin was used. Differentiated cells were identified based on typical morphology and their attachment to the growth plate surface within 2 days of initiation of differentiation [21]. The differentiated KG1 cells were trypsinized and used in the experiments. 

### 4.3. Determination of IL Levels and Cell Viability

IL-8 and IL-6 levels were determined as described previously [21] with the following modifications: For mono-cultures, A549 and KG1 cells were plated separately at 2.4 × 10^4^ cells per well in DMEM complete media (100 µL) in 96-well cell culture plates. For the co-cultures setup, we adjusted the number of A549 cells to differentiated KG1 at a 3:1 ratio [39]. A549 cells were plated at 1.2 × 10^4^ cells per well in DMEM complete media (100 µL) in a 96-well cell culture plate and were incubated in a humidified incubator overnight with complete DMEM. Subsequently, we added the differentiated KG1 cells (0.4 × 10^4^) cells per well on top of the A549 cells. Cells attached and incubated overnight in complete DMEM at 37 °C in air and 5% CO_2_ in a humidified incubator. Cell excitation was performed with 300 ng/mL TNFα and treatments were performed with FCBD:std, diclofenac, ibuprofen, dexamethasone, and budesonide in 100 µL complete DMEM. IL-8 and IL-6 secretion levels were analyzed after incubation of 4 h in both monocultures and co-cultures. Supernatant samples were collected and tested for IL-8 and IL-6 levels using IL-8 and IL-6 ELISA kits (DY208 and DY206, respectively, R&D Systems, Minneapolis, MN, USA). An Alamar Blue (resazurin) assay was performed to determine cell viability. For dose–response assays GraphPad Prism version 6.1 (https://www.graphpad.com/scientific-software/prism/ (accessed on 8 August 2021), GraphPad Software Inc., San Diego, CA, USA) was used to produce dose–response curves and IC50 doses were calculated using nonlinear regression analysis.

### 4.4. Analysis of Combined Drug Effects

Drug synergy was calculated based on ELISA results of A549 or KG1 cells by the Bliss independence drug interaction model expresses treating drug combinations in a non- interaction as joint action of independent, but competing interference by the individual drugs [40]. The response was measured as the percentage of interleukins secreted from cells following treatment in comparison to control. The Bliss-predicted combination response Exy is calculated by the following equation: Exy = Ex + Ey − (Ex × Ey); x and y are drugs; Ex or Ey are (1 − [fraction of interleukins secreted in drug/control]). Exy is the expected additive effect of the combined treatment with Ex and Ey. 0 ≤ Ei ≤ 1, i = x, y or xy. The synergy expressed as the delta of Exy and the observed effect of the combined treatment (x, y). The values differed on a scale of: >0 = synergism, 0 = independent, no synergy, <0 = antagonism [41]. Values are shown as mean. Statistical significant was determined by comparisons between more than 2 groups were made with analysis of variance (ANOVA) followed by Tukey–Kramer’s honest significant difference (HSD) test as post hoc. Different letters indicate significantly different levels of the experimental (observed) values from all combinations of pairs (*n* = 3; HSD; *p* ≤ 0.05).

### 4.5. Quantitative Real-Time PCR 

Quantitative real-time PCR (qPCR) was carried out as previously described [21]. Briefly, A549 cells were seeded in a 6-well plate at a concentration of 75 × 10^4^ cells in 5 mL of complete DMEM media per well, incubated overnight in a CO_2_ incubator. Later, the media was removed and 25 × 10^4^ macrophages in 1.5 mL of complete DMEM media were added per well on top of the A549 cells. After 24 h incubation cells were treated with TNFα, FCBD:std, diclofenac, ibuprofen, and their combinations, or vehicle control (methanol 0.2 to 0.8% and DMSO 0.5 to 1%) for 2 h Cells were then harvested and extraction of total RNA was done using TRI Reagent (T9424; Sigma Aldrich, St. Louis, MO, USA). Purification and concentration of extracted RNA were conducted using GeneJET RNA Cleanup and Concentration Micro Kit (Ref-K0841; Thermo Scientific, Waltham, MA, USA). RNA was reverse-transcribed using qPCRBIO cDNA Synthesis Kit (PB30.11-10; PCR Biosystems IncqPCRBIO, Wayne, PA, USA). Primers were designed and PCR was performed using qPCRBIO SyGreen Blue Mix Hi-ROX (PB20.16-05, PCR Biosystems) in a StepOnePlus system (AB4346906. Applied Biosystems, Thermo Fisher Scientific, Waltham, MA, USA). The expression of each target gene was normalized to the expression of β-actin mRNA using the 2^−ΔΔCt^ method as the differences (Δ) in threshold cycle (Ct) between the target gene and β-actin. ΔCt = Ct target gene − Ct β-actin. ΔΔCt = ΔCt treatment − ΔCt vehicle control. Experiments were repeated three times. 

The primers were, for human: β-actin (Gene ID:60) (forward) 5′-AGTACTCCGTGTGGATCGGC-3′ (reverse) 5′-GCTGATCCACATCTGCTGGA-3′; *CCL2* (Gene ID:6347) (forward) 5′-AGATCTGTGCTGACCCCAAG-3′ (reverse) 5′-TCTTGGGTTGTGGAGTGAGT-3′; *IL-6* (Gene ID:3569) (forward) 5′-TACCCCCAGGAGAAGATTCC-3′ (reverse) 5′-TTTTCTGCCAGTGCCTCTTT-3′; *IL-8* (Gene ID: 3576) (forward) 5′-CAG GAA TTG AAT GGG TTT GC -3′ (reverse) 5′-AAA CCA AGG CAC AGT GGA AC -3′; COX-2 (Gene ID: 4513) (forward) 5′-ATTGACCAGAGCAGGCAGAT-3′ (reverse)-5′-CAGGATACAGCTCCACAGCA -3′; COX-1 (Gene ID: 4512) (forward) -5′-TACTCACAGTGCGCTCCAAC -3′ (reverse) 5′-GCAACTGCTTCTTCCCTTTG -3′. 

### 4.6. D Models

Hydrogel (AGFCH) included the combination of alginate (38.5% *v*/*v*; W201502; Sigma-Aldrich, St. Louis, MI, USA), gelatin (38.5% *v*/*v*; G9764; Bio-Basic, Amherst, NY, USA), fibrinogen (7.7% *v*/*v*; F3879l; Sigma-Aldrich), collagen (7.7% *v*/*v*; C9791; Sigma-Aldrich) and hyaluronic acid (7.7% *v*/*v*; O8185; Sigma-Aldrich). Alginate was dissolved in 10% glycerol in PBS at stock concentrations of 19.6–25 mg/mL, gelatin was dissolved in 10% glycerol in PBS at stock concentrations of 45–75 mg/mL. Alginate and gelatin were mixed at a 1:1 ratio. Fibrinogen was dissolved in PBS at a stock concentration of 50 mg/mL, collagen was dissolved in 0.1 M acetic acid at a stock concentration of 2.2 mg/mL and hyaluronic acid was dissolved in water at a stock concentration of 2 mg/mL. The 3D model was prepared on ThinCert^TM^—24 well, PET membrane, 8.0 μm pore size (662638, Greiner bio-one). The hydrogel was mixed with 0.8 × 10^4^ differentiated KG1 cells per membrane and printed on the basal side of ThinCert. Cross-linking was done using 990 µL/mL CaCl2 (A610050; Bio-Basic) from the stock concentration of 50 mM and 10 U thrombin (SRP6557; Sigma-Aldrich) from stock concentration of 1000 U/mL. The cross-linking duration was 5 min.

The printed structure on the ThinCert was washed with PBS and transferred to a 24-well plate containing 300 µL complete DMEM growth media. 2.4 × 10^4^ A549 cells were added to the apical side of the ThinCert and allowed to grow as a monolayer for 2 days in complete DMEM growth media. The membrane of the ThinCert was removed using a sterile surgical blade and transferred to a 96-well plate. Treatments were performed for 4 h, and supernatant of samples was collected and tested for IL-8 and IL-6 levels using IL-8 and IL-6 ELISA kits. Structures were stained using EasyProbe Hoechst and Dil perchlorate membrane staining as described above. To assess cell viability in the 3D structure, they were washed with PBS and Alamar Blue assay (resazurin, AR002; R&D Systems, Minneapolis, MN, USA) was performed. 

### 4.7. Cellular Staining and Confocal Microscopy

The 3D model supported on the tissue culture inserts was washed with PBS, and the membrane was removed from the insert using a surgical blade. The membrane was washed with PBS and incubated with Dil perchlorate for membrane staining and Hoechst for nuclear staining for 40 min (C017 and -FP027, respectively; ABP Bioscience, Rockville, MD, USA). Cell microscopy and image acquisition were carried out using a Leica SP8 laser scanning microscope (Leica, Wetzlar, Germany) equipped with 405, 488 and 552 nm solid-state lasers, HCX PL APO CS 10x/0.40 or HC PL APO CS 63x/1.2 water immersion objectives (Leica, Wetzlar, Germany) and Leica Application Suite X software (LASX, Leica, Wetzlar, Germany). Hoechst, 5(6)-Carboxyfluorescein and membrane-emission signals were detected with PMT and HyD (hybrid) detectors in ranges of 415–490 nm, 500–535 nm and 565–660 nm, respectively.

### 4.8. Determination of COX Activity

Inhibition of COX activity was determined using Cyclooxygenase 1 (COX1) Inhibitor Screening Assay Kit (ab204698, Abcam, Cambridge, UK) and Cyclooxygenase 2 (COX2) Inhibitor Screening Assay Kit (K547-100, BioVisionInc., Milpitas, CA, USA). The fluorometric detection method was applied in 10 µL COX assay buffer, according to manufacturer instructions.

### 4.9. Statistical Analysis

The JMP statistical package (https://www.jmp.com/en_us/home.html, accessed on 10 August 2021) SAS Inc., Carrey, NC, USA) was used for data were processing. Comparisons between any two groups were done using the Student’s *t*-Test. Comparisons between more than 2 groups were done using analysis of variance (ANOVA) followed by Tukey–Kramer’s honest significant difference (HSD) test as post hoc. *p* values ≤ 0.05 were considered significant.

## 5. Conclusions

We have shown that FCBD:std and diclofenac have synergistic anti-inflammatory effects on macrophages and lung epithelial cells, which involve the reduction of *COX* and *CCL2* gene expression and IL levels. FCBD:std, when combined with diclofenac, can have considerably increased anti-inflammatory activity by several fold, suggesting that in an effective cannabis-diclofenac combined treatment, the level of NSAIDs may be reduced without compromising anti-inflammatory effectivity. It should be noted, however, that A549 and KG1 cells are immortalized lung carcinoma epithelial cells and macrophage derived from bone marrow myelogenous leukemia, respectively. Since cancer cell lines are known to deviate pharmacologically from in vivo or ex vivo testing, additional studies are needed on, e.g., ex vivo human lung tissue or alveolar organoids to verify the presented synergies. This combined activity of cannabis with NSAID needs to be examined also in clinical trials.

## Data Availability

Data is contained within the article and Supplementary Materials.

## Supplementary Materials

The following supporting information can be downloaded at: https://www.mdpi.com/article/10.3390/ph15121559/s1, Supplementary Figure S1. Relative inhibition level (%) of (a) COX-1 (b) COX-2 activity in the presence of FCBD:std, ibuprofen (Ibu) or diclofenac (Diclo). Vehicle control is 1% methanol (1% DMSO vehicle control is not significantly different from 1% methanol, not shown); Supplementary Figure S2. Structure of the 3D printed model. In green: 8 µm PEM membrane attached to the printed ECM. Designed and illustrated by Yoav Koltai; Supplementary Table S1a. IL-8 levels per cell relative, in percent, to vehicle (methanol 0.8%) + TNFα controls in differentiated KG1 cells and Fold change calculation of combined treatment; FCBD:std with ibuprofen (Ibu) in relation to ibuprofen only in the same concentration. Treatment duration was 4 h. Anti-inflammatory activity was determined by ELISA as a function of the level of IL-8 (pg/cell). Means ± standard error (SE, *n* = 3) are shown; Supplementary Table S1b. IL-8 levels per cell relative, in percent, to vehicle (methanol 0.2% and DMSO 0.5%) + TNFα controls in differentiated KG1 cells and Fold change calculation of combined treatment; FCBD:std with budesonide (Bud) in relation to budesonide only in the same concentration. Treatment duration was 4 h. Anti-inflammatory activity was determined by ELISA as a function of the level of IL-8 (pg/cell). Means ± standard error (SE, *n* = 3) are shown; Supplementary Table S1c. IL-8 levels per cell relative, in percent, to vehicle (methanol 0.2% and DMSO 0.5%) + TNFα controls in differentiated KG1 cells and Fold change calculation of combined treatment; FCBD:std with dexamethasone (Dexa) in relation to dexamethasone only in the same concentration. Treatment duration was 4 h. Anti-inflammatory activity was determined by ELISA as a function of the level of IL-8 (pg/cell). Means ± standard error (SE, *n* = 3) are shown; Supplementary Table S1d. IL-8 levels per cell relative, in percent, to vehicle (methanol 0.2% and DMSO 1%) + TNFα controls in differentiated KG1 cells and Fold change calculation of combined treatment; FCBD:std with diclofenac (Diclo) in relation to diclofenac only in the same concentration. Treatment duration was 4 h. Anti-inflammatory activity was determined by ELISA as a function of the level of IL-8 (pg/cell). Means ± standard error (SE, *n* = 3) are shown; Supplementary Table S2. Different letters signify delta values that are significantly different from all combinations of pairs according to the Tukey–Kramer honest significant difference test (HSD; *p* ≤ 0.05). Delta values calculated according to the Bliss model between the experimental (observed) and the calculated (expected) values of the synergistic interactions between FCBD:std with ibuprofen (a), budesonide (b), dexamethasone (c) or diclofenac (d) on anti-inflammatory activity of KG1 cells following combined treatments. Delta between observed and experimental values is graphically presented in Figure 1; Supplementary Table S3a. IL-8 levels per cell relative, in percent, to vehicle (methanol 0.8%) + TNFα controls in A549 cells and Fold change calculation of combined treatment; FCBD:std with ibuprofen (Ibu) in relation to ibuprofen only in the same concentration. Treatment duration was 4 h. Anti-inflammatory activity was determined by ELISA as a function of the level of IL-8 (pg/cell). Means ± standard error (SE, *n* = 3) are shown; Supplementary Table S3b. IL-8 levels per cell relative, in percent, to vehicle (methanol 0.2% and DMSO 0.5%) + TNFα controls in A549 cells and Fold change calculation of combined treatment; FCBD:std with budesonide (Bud) in relation to budesonide only in the same concentration. Treatment duration was 4 h. Anti-inflammatory activity was determined by ELISA as a function of the level of IL-8 (pg/cell). Means ± standard error (SE, *n* = 3) are shown; Supplementary Table S3c. IL-8 levels per cell relative, in percent, to vehicle (methanol 0.2% and DMSO 0.5%) + TNFα controls in A549 cells and Fold change calculation of combined treatment; FCBD:std with dexamethasone (Dexa) in relation to dexamethasone only in the same concentration. Treatment duration was 4 h. Anti-inflammatory activity was determined by ELISA as a function of the level of IL-8 (pg/cell). Means ± standard error (SE, *n* = 3) are shown; Supplementary Table S3d. IL-8 levels per cell relative, in percent, to vehicle (methanol 0.2% and DMSO 1%) + TNFα controls in A549 cells and Fold change calculation of combined treatment; FCBD:std with diclofenac (Diclo) in relation to diclofenac only in the same concentration. Treatment duration was 4 h. Anti-inflammatory activity was determined by ELISA as a function of the level of IL-8 (pg/cell). Means ± standard error (SE, *n* = 3) are shown; Supplementary Table S4. Different letters signify delta values that are significantly different from all combinations of pairs according to the Tukey–Kramer honest significant difference test (HSD; *p* ≤ 0.05). Delta values calculated according to the Bliss model between the experimental (observed) and the calculated (expected) values of the synergistic interactions between FCBD:std with ibuprofen (a), budesonide (b), dexamethasone (c) or diclofenac (d) on anti-inflammatory activity of A549 cells following combined treatments. The delta between observed and experimental values is graphically presented in Figure 2.
