# Supplementary material for: Phytocannabinoids Act Synergistically with Non-Steroidal Anti-Inflammatory Drugs Reducing Inflammation in 2D and 3D In Vitro Models
Source: Pharmaceuticals (Basel). 2022 Dec 14;15(12):1559. doi: 10.3390/ph15121559 (PMC9787964; doi:10.3390/ph15121559)
Supplement: Supplementary file 1 [file pharmaceuticals-15-01559-s001.zip › pharmaceuticals-2068987-supplementary.pdf]

Phytocannabinoids act synergistically with non-steroidal anti-inflammatory drugs reducing inflammation in 2D and 3D in vitro models

Ajjampura C Vinayaka[1], Nurit Shalev[1], Seegehalli M Anil[1], Sudeep Tiwari[1], Navin Kumar[1], Eduard Belausov[1], Karthik Ananth Mani [2,3], Guy Mechrez[2], Hinanit Koltai[1,2,*]

Supplementary Data

Figures

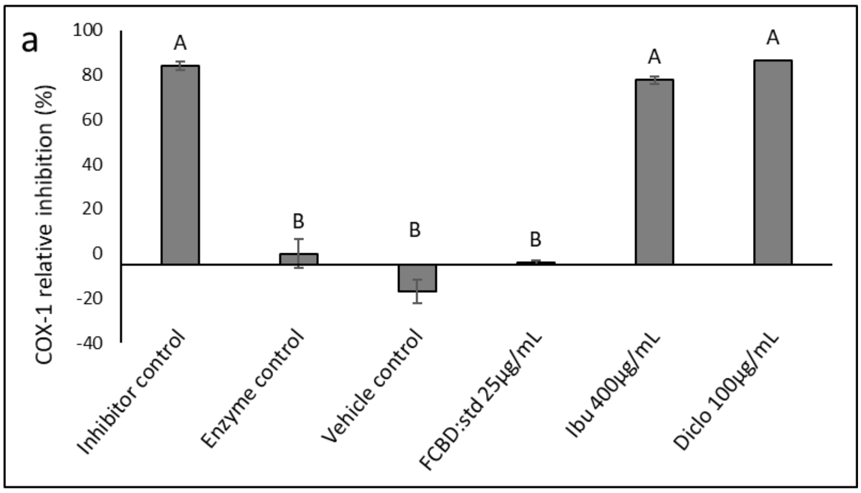

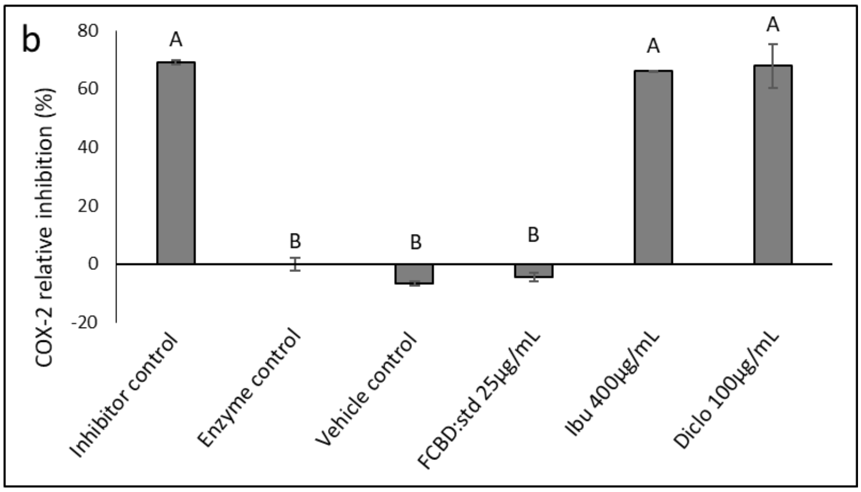

Supplementary Figure S1. Relative inhibition level (%) of (a) COX-1 (b) COX-2 activity in the presence of FCBD:std, ibuprofen (Ibu) or diclofenac (Diclo). Vehicle control is 1% methanol (1% DMSO vehicle control is not significantly different from 1% methanol, not shown).

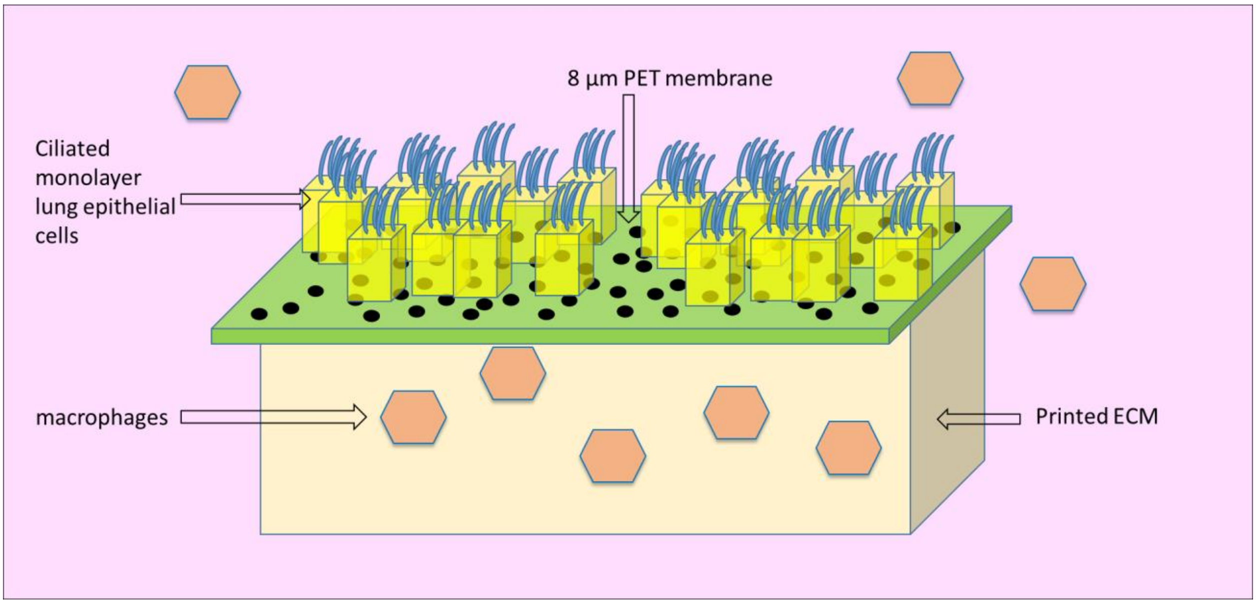

Supplementary Figure S2. Structure of the 3D printed model. In green: 8 µm PEM membrane attached to the printed ECM. Designed and illustrated by Yoav Koltai.

Tables

Treatments (all treated by TNFα)	IL-8 level per cell in treatment vs. vehicle + TNFα control (% mean±SE; n=3)	Fold change in activity vs. ibuprofen
Ibu 100 µg/mL	131.54±5.89	
Ibu 200 µg/mL	108.52±5.79	
Ibu 300 µg/mL	107.85±5.51	
Ibu 400 µg/mL	89.74±3.39	
Ibu 500 µg/mL	78.45±0.84	
FCBD:std 20 µg/mL	570.69±2.68	
FCBD:std 30 µg/mL	245.34±1.58	
FCBD:std 20 µg/mL + Ibu 100 µg/mL	490.01±75.75	0.3

FCBD:std 20 μg/mL + Ibu 200 μg/mL	147.21±9.51	0.7
FCBD:std 20 μg/mL + Ibu 300 μg/mL	29.75±6.46	3.6
FCBD:std 20 μg/mL + Ibu 400 μg/mL	25.28±3.60	3.6
FCBD:std 20 μg/mL + Ibu 500 μg/mL	13.71±1.82	5.7
FCBD:std 30 μg/mL + Ibu 100 μg/mL	65.44±3.98	2.0
FCBD:std 30 μg/mL + Ibu 200 μg/mL	78.98±6.46	1.4
FCBD:std 30 μg/mL + Ibu 300 μg/mL	46.87±11.67	2.3
FCBD:std 30 μg/mL + Ibu 400 μg/mL	25.91±3.93	3.5

Supplementary Table S1a. IL-8 levels per cell relative, in percent, to vehicle (methanol 0.8%)+TNFα controls in differentiated KG1 cells and Fold change calculation of combined treatment; FCBD:std with ibuprofen (Ibu) in relation to ibuprofen only in the same concentration. Treatment duration was 4 h. Anti-inflammatory activity was determined by ELISA as a function of the level of IL-8 (pg/cell). Means ± standard error (SE, n=3) are shown.

Treatments (all treated by TNFα)	IL-8 level per cell in treatment vs. vehicle + TNFα control (% mean±SE; n=3)	Fold change in activity vs. budesonide
Bud 50 ng/mL	66.82±1.80	
Bud 100 ng/mL	66.83±8.22	
Bud 250 ng/mL	72.72±5.33	
Bud 500 ng/mL	55.54±4.09	
Bud 1000 ng/mL	54.11±1.60	

FCBD:std 30 μg/mL	140.32±10.26	
FCBD:std 30 μg/mL + Bud 50 ng/mL	33.73±5.23	2.0
FCBD:std 30 μg/mL + Bud 100 ng/mL	65.32±6.70	1.0
FCBD:std 30 μg/mL + Bud 250 ng/mL	62.98±2.58	1.2
FCBD:std 30 μg/mL + Bud 500 ng/mL	75.52±5.64	0.7
FCBD:std 30 μg/mL + Bud 1000 ng/mL	57.18±18.36	0.9

Supplementary Table S1b. IL-8 levels per cell relative, in percent, to vehicle (methanol 0.2% and DMSO 0.5%)+TNFα controls in differentiated KG1 cells and Fold change calculation of combined treatment; FCBD:std with budesonide (Bud) in relation to budesonide only in the same concentration. Treatment duration was 4 h. Anti-inflammatory activity was determined by ELISA as a function of the level of IL-8 (pg/cell). Means ± standard error (SE, n=3) are shown.

Treatments (all treated by TNFα)	IL-8 level per cell in treatment vs. vehicle + TNFα control (% mean± SE; n=3)	Fold change in activity vs. dexamethasone
Dexa 250 ng/mL	82.04±6.30	
Dexa 500 ng/mL	101.36±28.93	
Dexa 1000 ng/mL	71.0±0.80	
Dexa 2000 ng/mL	77.316±11.95	
Dexa 4000 ng/mL	65.876±2.53	
FCBD:std 30 μg/mL	130.936±10.40	
FCBD:std 30 μg/mL + Dexa 250 ng/mL	25.486±1.39	3.2
FCBD:std 30 μg/mL + Dexa 500 ng/mL	36.90±11.64	2.7

FCBD:std 30 μg/mL + Dexa 1000 ng/mL	24.31±9.64	2.9
FCBD:std 30 μg/mL + Dexa 2000 ng/mL	20.85±14.65	3.7

Supplementary Table S1c. IL-8 levels per cell relative, in percent, to vehicle (methanol 0.2% and DMSO 0.5%)+TNFα controls in differentiated KG1 cells and Fold change calculation of combined treatment; FCBD:std with dexamethasone (Dexa) in relation to dexamethasone only in the same concentration. Treatment duration was 4 h. Anti-inflammatory activity was determined by ELISA as a function of the level of IL-8 (pg/cell). Means ± standard error (SE, n=3) are shown.

Treatments (all treated by TNFα)	IL-8 level per cell in treatment vs. vehicle + TNFα control (% mean±SE; n=3)	Fold change in activity vs. Diclofenac
Diclo 5 μg/mL	117.80±6.35	
Diclo 25 μg/mL	95.26±2.16	
Diclo 50 μg/mL	89.29±2.53	
Diclo 75 μg/mL	76.02±1.99	
Diclo 100 μg/mL	74.37±3.19	
FCBD:std 20 μg/mL	318.04±14.08	
FCBD:std 30 μg/mL	347.11±2.66	
FCBD:std 20 μg/mL + Diclo 50 μg/mL	163.03±3.78	0.5
FCBD:std 20 μg/mL + Diclo 75 μg/mL	92.29±2.45	0.8
FCBD:std 20 μg/mL + Diclo 100 μg/mL	76.48±1.45	1.0
FCBD:std 30 μg/mL + Diclo 5μg/mL	296.57±12.83	0.4
FCBD:std 30 μg/mL + Diclo 25 μg/mL	228.66±6.32	0.4

FCBD:std 30 μg/mL + Diclo 50 μg/mL	72.64±3.45	1.2
FCBD:std 30 μg/mL + Diclo 75 μg/mL	37.24±1.89	2.0
FCBD:std 30 μg/mL + Diclo 100 μg/mL	39.27±1.51	1.9

Supplementary Table S1d. IL-8 levels per cell relative, in percent, to vehicle (methanol 0.2% and DMSO 1%)+TNFα controls in differentiated KG1 cells and Fold change calculation of combined treatment; FCBD:std with diclofenac (Diclo) in relation to diclofenac only in the same concentration. Treatment duration was 4 h. Anti-inflammatory activity was determined by ELISA as a function of the level of IL-8 (pg/cell). Means ± standard error (SE, n=3) are shown.

		FCBD:std (μg/mL)					
		0	5	10	20	30	40
a	0	E	E	E	E	E	E
	100	E	E	E	C	C	E
	200	E	E	E	AB	C	DE
Ibuprofen (μg/mL)	300	E	E	E	A	C	E
	400	E	E	E	AB	C	E
	500	E	E	DE	B	CD	E
		FCBD:std (μg/mL)					
		0	5	10	20	25	30
b	0	ABCDE	ABCDE	ABCDE	ABCDE	ABCDE	ABCDE
	50	ABCDE	ABCDE	ABCDEF	ABCDEF	DEF	A
	100	ABCDE	ABCDEF	ABCDEF	ABCDEF	ABCDEF	ABC
Budesonide (ng/mL)	250	ABCDE	ABCDEF	EF	ABCDEF	BCDEF	AB
	500	ABCDE	ABCDEF	CDEF	EF	F	ABCDE
	1000	ABCDE	ABCDEF	BCDEF	DEF	ABCDEF	ABCD
		FCBD:std (μg/mL)					
		0	5	10	20	25	30
c	0	ABC	ABC	ABC	ABC	ABC	ABC
	250	ABC	ABC	DE	F	AB	A

	500	ABC	ABC	DE	F	BC	AB
Dexamethasone (ng/mL)	1000	ABC	ABC	DE	DEF	A	AB
	2000	ABC	ABC	DEF	EF	CD	AB
	4000	ABC	ABC	DE	EF	AB	A

d		FCBD:std (μg/mL)					
		0	5	10	20	30	40
Diclofenac (μg/mL)	0	G	G	G	G	G	G
	5	G	G	G	EF	CD	G
	25	G	G	G	G	DE	G
	50	G	G	G	CD	A	FG
	75	G	G	G	BC	A	G
	100	G	G	G	B	A	G

Supplementary Table S2. Different letters signify delta values that are significantly different from all combinations of pairs according to the Tukey–Kramer honest significant difference test (HSD; $P \leq 0.05$). Delta values calculated according to the Bliss model between the experimental (observed) and the calculated (expected) values of the synergistic interactions between FCBD:std with ibuprofen (a), budesonide (b), dexamethasone (c) or diclofenac (d) on anti-inflammatory activity of KG1 cells following combined treatments. Delta between observed and experimental values is graphically presented in Figure 1.

Treatments (all treated by TNFα)	IL-8 level per cell in treatment vs. vehicle + TNFα control (% mean±SE; n=3)	Fold change in activity vs. ibuprofen
FCBD:std 20 μg/mL	95.79±2.32	
Ibu 300 μg/mL	110.46±2.80	
Ibu 400 μg/mL	104.41±5.89	
Ibu 500 ug/mL	98.30±1.12	
FCBD:std 20 μg/mL + Ibu 300 μg/mL	72.69±1.84	1.5

FCBD:std 20 μg/mL + Ibu 400 μg/mL	50.97±1.70	2.0
FCBD:std 20 μg/mL + Ibu 500 μg/mL	44.65±4.10	2.2

Supplementary Table S3a. IL-8 levels per cell relative, in percent, to vehicle (methanol 0.8%)+TNFα controls in A549 cells and Fold change calculation of combined treatment; FCBD:std with ibuprofen (Ibu) in relation to ibuprofen only in the same concentration. Treatment duration was 4 h. Anti-inflammatory activity was determined by ELISA as a function of the level of IL-8 (pg/cell). Means ± standard error (SE, n=3) are shown.

Treatments (all treated by TNFα)	IL-8 level per cell in treatment vs. vehicle + TNFα control (% mean±SE; n=3)	Fold change vs. to budesonide
Bud 50 ng/mL	98.71±0.99	
Bud 100 ng/mL	105.00±1.34	
FCBD:std 5 μg/mL	90.50±0.87	
FCBD:std 10 μg/mL	96.81±1.40	
FCBD:std 20 μg/mL	82.71±0.28	
FCBD:std 5 μg/mL + Bud 100 ng/mL	57.32±2.83	1.8
FCBD:std 10 μg/mL + Bud 50 ng/mL	65.77±1.06	1.5
FCBD:std 10 μg/mL + Bud 100 ng/mL	66.25±2.19	1.6
FCBD:std 20 μg/mL + Bud 100 ng/mL	53.79±0.85	2.0

Supplementary Table S3b. IL-8 levels per cell relative, in percent, to vehicle (methanol 0.2% and DMSO 0.5%)+TNFα controls in A549 cells and Fold change calculation of combined treatment; FCBD:std with budesonide (Bud) in relation to budesonide only in the same concentration. Treatment duration was 4 h. Anti-inflammatory activity was determined by ELISA as a function of the level of IL-8 (pg/cell). Means ± standard error (SE, n=3) are shown.

Treatments (all treated by TNFα)	IL-8 level per cell in treatment vs. vehicle + TNFα control (% mean±SE; n=3)	Fold change in activity vs. dexamethasone
Dexa 250 ng/ mL	64.66±1.14	
Dexa 500 ng/mL	60.51±1.69	
Dexa 1000 ng/mL	59.77±0.97	
Dexa 2000 ng/mL	63.46±6.59	
Dexa 4000 ng/mL	64.84±4.31	
FCBD:std 10μg/mL	116.98±4.28	
FCBD:std 10 μg/mL + Dexa 250 ng/mL	60.97±0.31	1.1
FCBD:std 10 μg/mL + Dexa 500 ng/mL	58.07±2.42	1.0
FCBD:std 10 μg/mL + Dexa 1000 ng/mL	48.46±0.96	1.2
FCBD:std 10 μg/mL + Dexa 2000 ng/mL	59.13±4.59	1.1
FCBD:std 10 μg/mL + Dexa 4000 ng/mL	56.39±2.11	1.1

Supplementary Table S3c. IL-8 levels per cell relative, in percent, to vehicle (methanol 0.2% and DMSO 0.5%)+TNFα controls in A549 cells and Fold change calculation of combined treatment; FCBD:std with dexamethasone (Dexa) in relation to dexamethasone only in the same concentration. Treatment duration was 4 h. Anti-inflammatory activity was determined by ELISA as a function of the level of IL-8 (pg/cell). Means ± standard error (SE, n=3) are shown.

Treatments (all treated by TNFα)	IL-8 level per cell in treatment vs. vehicle + TNFα control (% mean±SE; n=3)	Fold change in activity vs. diclofenac
FCBD:std 5 μg/mL	104.81±8.13	
FCBD:std-10 μg/mL	104.88±8.45	
Diclo 50 μg/mL	101.42±1.08	

Diclo 100 μg/mL	94.20±1.37	
Diclo 200 μg/mL	87.45±1.74	
Diclo 300 μg/mL	67.56±0.81	
FCBD:std 5 μg/mL + Diclo 50 μg/mL	74.45±2.69	1.4
FCBD:std 10 μg/mL + Diclo 50 μg/mL	77.82±4.21	1.3
FCBD:std 5 μg/mL + Diclo 100 μg/mL	62.29±3.34	1.5
FCBD:std 5 μg/mL+Diclo 200 μg/mL	47.34±0.20	1.8
FCBD:std 5 μg/mL+Diclo 300 μg/mL	45.49±2.76	1.5
FCBD:std 10 μg/mL+Diclo 200 μg/mL	63.67±3.53	1.4

Supplementary Table S3d. IL-8 levels per cell relative, in percent, to vehicle (methanol 0.2% and DMSO 1%)+TNFα controls in A549 cells and Fold change calculation of combined treatment; FCBD:std with diclofenac (Diclo) in relation to diclofenac only in the same concentration. Treatment duration was 4 h. Anti-inflammatory activity was determined by ELISA as a function of the level of IL-8 (pg/cell). Means ± standard error (SE, n=3) are shown.

		FCBD:std (μg/mL)					
		0	5	10	20	30	40
a	0	DEFGH	DEFGH	DEFGH	DEFGH	DEFGH	DEFGH
	100	DEFGH	HI	BCDEFGH	CDEFGH	BCDE	BCDEFG
	200	DEFGH	HI	BCDEFG	BCDE	ABCD	BCDEF
Ibuprofen (μg/mL)	300	DEFGH	GHI	BCDE	AB	ABC	BCDEFG H
	400	DEFGH	FGHI	EFGHI	A	ABCD	DEFGH
	500	DEFGH	CDEFGH	BCDE	A	BCDEF	I

		FCBD:std (μg/mL)					
		0	5	10	20	30	40
b	0	CDEFGH I	CDEFGH I	CDEFGHI	CDEFGHI	CDEFGH I	CDEFGHI
Budosonide (ng/mL)	50	CDEFGH I	ABCD	ABC	ABCDE	DEFGHI	EFGHI

	100	CDEFGHI	A	A	AB	CDEFGH	EFGHI
	250	CDEFGHI	ABCDEF	ABCDEFG	BCDEFGH	GHI	FGHI
	500	CDEFGHI	ABCDEF	ABCDEFGH	BCDEFGH	FGHI	HI
	1000	CDEFGHI	DEFGHI	BCDEFGH	EFGHI	HI	I

		FCBD:std (µg/mL)					
		0	5	10	20	30	40
c	0	CD	CD	CD	CD	CD	CD
	250	CD	CD	ABC	D	ABCD	ABCD
	500	CD	ABCD	ABCD	D	BCD	ABCD
Dexamethasone (ng/mL)	1000	CD	ABCD	A	D	ABCD	ABCD
	2000	CD	ABCD	ABC	BCD	ABCD	BCD
	4000	CD	ABCD	AB	BCD	ABCD	ABCD

		FCBD:std (µg/mL)					
d		0	5	10	20	25	30
	0	DEFGH	DEFGH	DEFGH	DEFGH	DEFGH	DEFGH
	50	DEFGH	ABC	ABC	ABCD	BCDE	HIJKL
	100	DEFGH	AB	CDEFG	BCDE	BCDE	HIJKL
Diclofenac (µg/mL)	200	DEFGH	A	ABC	BCDE	CDEFG	HIJKL
	300	DEFGH	ABC	CDEFG	EFGHI	HIJKL	JKLMN
	400	DEFGH	BCDEF	EFGHI	GHIJK	KLMN	LMN

Supplementary Table S4. Different letters signify delta values that are significantly different from all combinations of pairs according to the Tukey–Kramer honest significant difference test (HSD; $P \leq 0.05$). Delta values calculated according to the Bliss model between the experimental (observed) and the calculated (expected) values of the synergistic interactions between FCBD:std with ibuprofen (a), budesonide (b), dexamethasone (c) or diclofenac (d) on anti-inflammatory activity of A549 cells following combined treatments. The delta between observed and experimental values is graphically presented in Figure 2.
